# Longitudinal Analysis of Antibody Response Following SARS-CoV-2 Infection Depending on Disease Severity: A Prospective Cohort Study

**DOI:** 10.3390/v15112250

**Published:** 2023-11-13

**Authors:** Christina Zirou, Sentiljana Gumeni, Ioannis Bellos, Ioannis Ntanasis-Stathopoulos, Aimilia D. Sklirou, Tina Bagratuni, Eleni Korompoki, Filia Apostolakou, Ioannis Papassotiriou, Ioannis P. Trougakos, Evangelos Terpos

**Affiliations:** 1 Department of Internal Medicine, Sotiria General and Chest Diseases Hospital of Athens, 11527 Athens, Greece; 2 Department of Cell Biology and Biophysics, Faculty of Biology, National and Kapodistrian University of Athens, 15771 Athens, Greece; 3 Department of Hygiene, Epidemiology and Medical Statistics, School of Medicine, National and Kapodistrian University of Athens, 11527 Athens, Greece; 4 Department of Clinical Therapeutics, School of Medicine, National and Kapodistrian University of Athens, 11528 Athens, Greece; 5 Department of Clinical Biochemistry, “Aghia Sophia” Children’s Hospital, 11527 Athens, Greece

**Keywords:** COVID-19, SARS-CoV-2, severity, antibody, immune system, vaccination

## Abstract

Objective: Severe coronavirus disease 19 (COVID-19) is characterized by a dysregulated inflammatory response, with humoral immunity playing a central role in the disease course. The objective of this study was to assess the immune response and the effects of vaccination in recovered individuals with variable disease severity up to one year following natural infection. Methods: A prospective cohort study was conducted including patients with laboratory-confirmed COVID-19. Disease severity was classified as mild, moderate, and severe based on clinical presentation and outcomes. Anti-RBD (receptor binding domain) and neutralizing antibodies were evaluated at multiple timepoints during the first year after COVID-19 diagnosis. Results: A total of 106 patients were included; of them, 28 were diagnosed with mild, 38 with moderate, and 40 with severe disease. At least one vaccine dose was administered in 58 individuals during the follow-up. Participants with mild disease presented significantly lower anti-RBD and neutralizing antibodies compared to those with moderate and severe disease up to the 3rd and 6th months after the infection, respectively. After adjusting for covariates, in the third month, severe COVID-19 was associated with significantly higher anti-RBD (β: 563.09; 95% confidence intervals (CI): 257.02 to 869.17) and neutralizing (β: 21.47; 95% CI: 12.04 to 30.90) antibodies. Among vaccinated individuals, at the 12th month, a history of moderate disease was associated with significantly higher anti-RBD levels (β: 5615.19; 95% CI: 657.92 to 10,572.46). Conclusions: Severe COVID-19 is associated with higher anti-RBD and neutralizing antibodies up to 6 months after the infection. Vaccination of recovered patients is associated with a remarkable augmentation of antibody titers up to one year after COVID-19 diagnosis, regardless of disease severity.

## 1. Introduction

Coronavirus disease 19 (COVID-19), caused by the beta-coronavirus SARS-CoV-2 (severe acute respiratory syndrome coronavirus 2), has rapidly emerged as a pandemic, threatening the integrity of healthcare systems worldwide. COVID-19 may range from silent and asymptomatic to lethal disease, as it may be complicated by serious pneumonia with respiratory failure, thrombosis, sepsis, or liver and kidney disease [1]. The development and widespread use of COVID-19 vaccines have managed to decrease the severity of the disease, although waning immunity [2] along with the emergence of new SARS-CoV-2 variants able to escape from vaccine-induced antibodies, such as the Omicron strain [3], raise concerns about long-term protection [4,5]. Therefore, research focusing on a deeper understanding of COVID-19 immunology has become a global priority [6].

Several risk factors have been proposed to predispose people to serious COVID-19 [7], especially increasing age, male sex, and metabolic syndrome [8,9]. The pathophysiology of severe disease may be based on the inability of the host to activate an effective immune response early in the course of the infection, possibly due to the presence of high viral load in conjunction with deficient antigen presentation and delayed interferon response. The lack of a timely immune response results in prolonged tissue damage and overactivation of the immune system, reflected by enhanced myelopoiesis, production of autoantibodies, and vascular damage [10]. Hence, it has been suggested that severe COVID-19 is characterized by a dysregulated inflammatory response, with hyperactivation of neutrophils, macrophages, and Th2 and Th17 cells as well as hypoactivation of dendritic, natural killer, Th1, and Treg cells [11,12].

Humoral immune response plays a crucial role in the course of COVID-19 [13], as reflected by the protective effects of both convalescent plasma administration in naïve hosts and the therapeutic effect of synthetically designed monoclonal antibodies [14]. Specifically, B-cells exert their action by producing neutralizing antibodies that block viral cell entry, as well as by antibody-dependent cellular cytotoxicity and T-cell activation [15]. Observational evidence has suggested that severe COVID-19 may elicit a stronger antibody response [16], although the durability of antibody levels and the effects of subsequent vaccination remain under investigation [17]. The present prospective study aims to confirm the potential association between the severity of SARS-CoV-2 infection and the elicited immune response, as well as to assess the kinetics of antibody titers in recovered individuals, before and after subsequent vaccination.

## 2. Materials and Methods

### 2.1. Study Design

This is a prospective cohort study aiming to assess the antibody response of adults infected by SARS-CoV-2, depending on disease severity. Individuals with laboratory-confirmed SARS-CoV-2 infection presenting with COVID-19 symptoms at the emergency department of Sotiria General Hospital of Chest Diseases from January to May 2021 were consecutively enrolled. Sotiria General Hospital of Chest Diseases served as the largest COVID-19 reference hospital during the first SARS-CoV-2 waves, admitting patients at risk of progression to severe COVID-19. Laboratory diagnosis was verified by real-time polymerase chain reaction (RT-PCR) analysis of nasopharyngeal swabs. Pediatric patients and individuals considered unable to provide written informed consent were excluded. The study was approved by the institutional review board of the hospital and was conducted in accordance with the Declaration of Helsinki and the International Conference on Harmonization for Good Clinical Practice.

### 2.2. Data Collection

Variables regarding demographics, comorbidities, clinical presentation, treatment, and clinical outcomes were registered in pre-specified forms. The Pneumonia Severity Index/Pneumonia Outcome Research Trial (PSI/PORT) score was calculated as an index of pneumonia severity [18]. The comorbidities of patients were summarized by estimating the Charlson Comorbidity Index [19]. Information about vaccination against SARS-CoV-2 following the infection (vaccine type, number of doses, and interval from infection) was also collected. Patients were classified into 3 categories (mild, moderate, and severe) depending on the severity of COVID-19. Specifically, mild cases were characterized by a lack of signs of clinically important pneumonia and need for oxygen therapy. Moderate disease referred to the need for hospitalization due to the presence of signs indicative of COVID-19 pneumonia such as fever, cough, and dyspnea in conjunction with lung infiltrates on chest computerized tomography (CT). Patients with moderate disease required, by definition, low-flow oxygen therapy delivered by nasal cannula. On the other hand, severe disease was defined by the presence of progressive respiratory failure with higher oxygen requirements (inspiratory oxygen fraction ≥ 40%), and/or by the diagnosis of acute respiratory distress syndrome (ARDS) and sepsis/septic shock, as well as the need for intensive care unit (ICU) admission.

Participants were followed for 1 year and blood samples were collected with venipuncture at the following pre-specified timepoints: 1st day (baseline), 7th day, 1st month, 3rd month, 6th month, and 12th month.

### 2.3. Measurement of Immune Responses

The methodology of anti-spike receptor binding domain protein IgG (anti-RBD) and neutralizing antibodies measurement has been described elsewhere [20]. In brief, the measurement of anti-RBD antibodies was performed using the Elecsys Anti-SARS-CoV-2 Assay (Roche Diagnostics GmbH, Mannheim, Germany), while neutralizing antibodies against SARS-CoV-2 were measured using the FDA-approved cPass™ SARS-CoV-2 NAbs Detection Kit (GenScript, Piscataway, NJ, USA), which allows the indirect detection of potential SARS-CoV-2 neutralizing antibodies in blood by testing antibody-mediated inhibition of SARS-CoV-2 RBD binding to the human host receptor angiotensin-converting enzyme 2.

### 2.4. Statistical Analysis

Data analysis was performed in R-3.6.5. A two-sided *p*-value threshold of 0.05 was used to define statistical significance. The distribution of continuous variables was visually assessed by the inspection of histograms. Variables were described by their median and interquartile range, and comparisons were performed using the non-parametric Mann–Whitney U test and the Kruskal–Wallis test. Categorical variables were compared across groups using the chi-square test or Fisher’s exact test, in case the assumptions of the first were not met [21]. Positivity thresholds were applied for anti-RBD antibodies according to the manufacturer’s instructions and for neutralizing antibodies (>30%) according to previous literature in the field [20]. A linear regression analysis was conducted to evaluate the association of disease severity with anti-RBD and neutralizing antibodies at different timepoints, before and after vaccination. Both univariate and adjusted models were fitted, adjusting for the following pre-defined parameters that were presumed to serve as important potential confounders: age, sex, ethnicity, obesity, Charlson Comorbidity Index, vaccine type, number of vaccine doses, and interval from vaccination.

## 3. Results

### 3.1. Baseline Characteristics

The present cohort included a total of 106 patients infected by SARS-CoV-2. Of them, 28 presented signs of mild disease, 38 were diagnosed with moderate disease, and 40 with severe disease (Table 1). The median age of the study population was 52 years (interquartile range (IQR): 42.3–59), and the majority of patients (67%) were males. The most common comorbidity was dyslipidemia (27.4%), followed by hypertension (22.6%) and diabetes mellitus (14.2%). Severe disease was significantly associated with older age, diabetes mellitus, obesity, higher Charlson Comorbidity Index, and history of smoking. Patients with severe COVID-19 presented more commonly with fever and dyspnea and had significantly higher PSI-PORT values at admission. Remdesivir and dexamethasone were administered in the majority of patients with severe disease. Six patients with severe COVID-19 pneumonia were admitted to the ICU, four were mechanically ventilated, and two of them died. No breakthrough infections were observed during the follow-up.

During the follow-up period, 58 patients received at least one dose of SARS-CoV-2 vaccine. Two vaccine doses were administered to eight patients. The most commonly used vaccine was the BNT162b2 one, and the median time from infection to vaccination was 6.4 months (IQR: 5.7–6.8). No significant differences were noted across the disease severity groups regarding the type of vaccine, number of doses, and interval from infection to vaccination.

### 3.2. Antibody Levels before Vaccination

**Figure 1:**
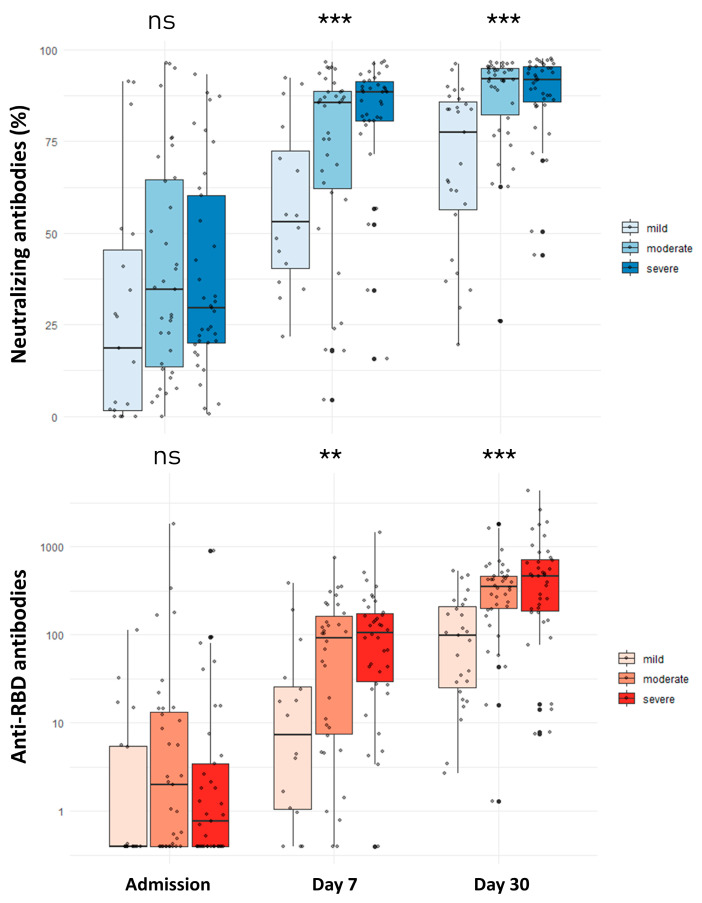
Comparison of neutralizing and anti-RBD antibodies among patients with mild, moderate, and severe COVID-19 at admission and days 7 and 30 after infection. ns: non-significant; ** *p*-value < 0.01; *** *p*-value < 0.001. ns: not significant Comparisons were performed with the Kruskal–Wallis test.

**Figure 2:**
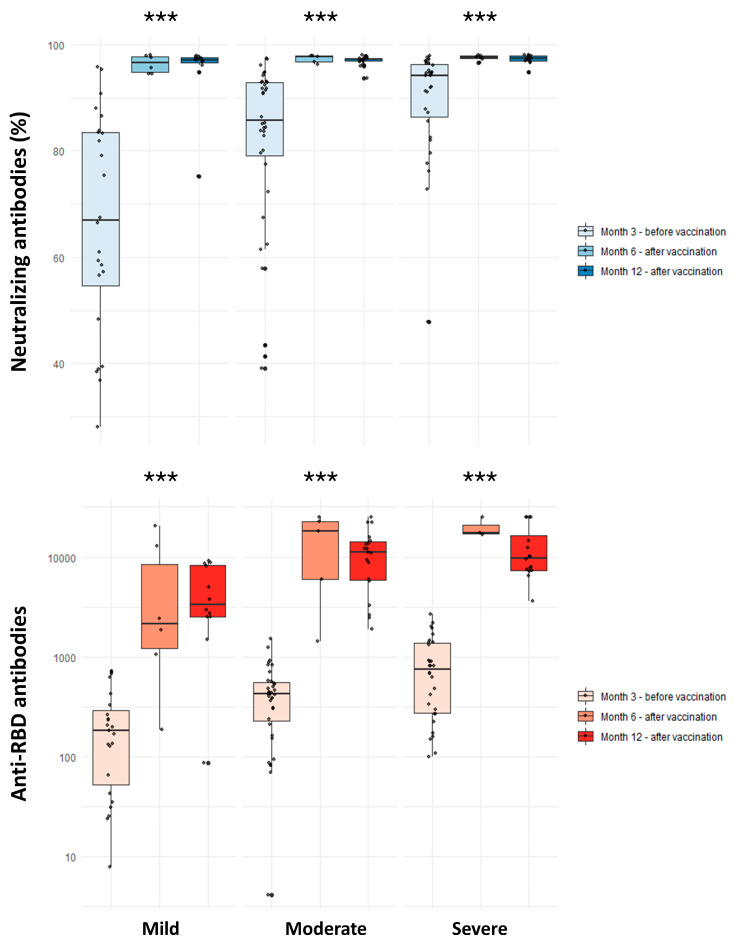
Comparison of neutralizing and anti-RBD antibodies among individuals with history of mild, moderate, and severe COVID-19 in the 3rd month (unvaccinated) and 6th and 12th months (vaccinated) following natural infection. ns: non-significant; *** *p*-value < 0.001. Comparisons were performed with the Kruskal –Wallis test.

At admission, the titer of anti-RBD antibodies was low (0.73 U/mL; IQR: 0.40–8.38) and similar across the disease severity groups (*p*-value: 0.160) (Figure 1). Specifically, 48.9% of participants had an anti-RBD titer > 0.8 U/mL (positivity threshold), while the percentage of positivity did not differ among those with mild (33.3%), moderate (57.1%), and severe disease (48.6%) (*p*-value: 0.260). Anti-RBD antibodies showed a significant rise starting from day 7 (67.62 U/mL; IQR: 8.49–162.23) up to the third month (419.40 U/mL; IQR: 169.30–711.50) after the infection (Figure 1 and Figure 2). Participants with mild disease presented significantly lower anti-RBD antibody values compared to those with both moderate and severe disease on the 7th day and the 1st and 3rd months after infection (Table 2). Patients with severe COVID-19 had significantly higher anti-RBD antibody levels than those with moderate disease only in the third month (747.05 vs. 426.95; *p*-value: 0.019). Regarding measurement at the 6th and 12th months after infection, no significant differences in anti-RBD antibodies were noted across the different groups for participants that remained unvaccinated. The outcomes of the linear regression analysis are presented in Table 3. After taking into account covariates, compared to mild disease, severe COVID-19 was associated with significantly higher anti-RBD antibody titers at the 1st (*β*: 427.35; 95% CI: 95.32 to 759.37) and 3rd (*β*: 563.09; 95% CI: 257.02 to 869.17) months.

Concerning neutralizing antibodies, no significant differences were observed across the three groups at admission (*p*-value: 0.190) (Figure 1). Overall, 47.3% of individuals had positive neutralizing antibodies (positivity threshold >30%), and this percentage did not differ significantly among those with mild (36.8%), moderate (51.4%), or severe (48.6%) disease (*p*-value: 0.577). The titer of neutralizing antibodies significantly increased at the seventh day and remained high up to the sixth month after infection (Figure 1 and Figure 2). Mild disease was associated with a significantly weaker neutralizing antibody response on the 7th day and the 1st, 3rd, and 6th months (Table 2). Patients with moderate disease presented a significantly lower neutralizing antibody titer than those with severe disease only in the third month after the initial infection (94.21% vs. 85.80%; *p*-value: 0.009). At the 12th month post-infection, no significant differences in neutralizing antibody titers were noted for patients with mild, moderate, and severe disease that remained unvaccinated. It should be noted that neutralizing antibody titer at 12 months was available in only one patient with mild disease without vaccination, while no significant difference was observed when patients with moderate and severe disease were compared (Mann–Whitney U test *p*-value: 0.057).

After adjusting for covariates, compared to mild disease, severe COVID-19 was associated with significantly higher neutralizing antibody titers 7 days after infection (*β*: 23.02; 95% CI: 8.17 to 37.88). In addition, in the fully adjusted model, the titer of neutralizing antibodies measured at the first month was significantly higher in patients with moderate (*β*: 13.87; 95% CI: 6.05 to 21.70) and severe (*β*: 18.26; 95% CI: 9.87 to 26.65) disease. Similarly, in the third month, stronger neutralizing antibody responses were found for patients with moderate (*β*: 12.44; 95% CI: 3.96 to 20.92) and severe (*β*: 21.47; 95% CI: 12.04 to 30.90) COVID-19 (Table 3).

### 3.3. Antibody Levels after Vaccination

The results of antibody measurements in participants that received at least one vaccine dose are shown in Table 2. Vaccination was linked to significantly higher anti-RBD antibody titer both at 6 (14,858 vs. 418 U/mL; *p*-value < 0.001) and 12 (8800 vs. 519 U/mL; *p*-value <0.001) months after infection. Vaccinated individuals with a history of mild disease developed a significantly weaker anti-RBD antibody response one year after SARS-CoV-2 infection as compared to those with moderate (3379 vs. 11,107 U/mL; *p*-value: 0.007) and severe (3379 vs. 9816 U/mL; *p*-value: 0.007) disease. In the age- and sex-adjusted model of vaccinated individuals, mild disease was significantly associated with lower anti-RBD antibody titers at the 12th month compared to moderate and severe COVID-19. In the fully adjusted model, this association remained significant only for moderate disease (*β*: 5615.19; 95% CI: 657.92 to 10,572.46) (Table 4). Regarding neutralizing antibodies, vaccination was associated with significantly higher titers at both 6 (97.59% vs. 80.80%; *p*-value <0.001) and 12 (97.32% vs. 73.35%; *p*-value < 0.001) months. No significant differences were observed in neutralizing antibody titers after vaccination across the disease severity groups (Table 2). Furthermore, no significant associations between COVID-19 severity and post-vaccination neutralizing antibodies were found in univariate and adjusted linear regression models (Table 4).

## 4. Discussion

The present study indicates that severe COVID-19 is associated with a stronger humoral immune response against SARS-CoV-2 compared to mild disease, starting early in the course of the disease and persisting at least up to 3 months after the initial infection. This effect was true for both anti-RBD and neutralizing antibodies and remained statistically significant after considering covariates that could affect antibody production, such as demographics, obesity, and comorbidities. Vaccination was linked to a remarkable immune response boost, leading to a 30-fold augmentation of anti-RBD antibodies and to consistently high neutralizing antibody levels of >97% up to 1 year after the infection. Interestingly, compared to moderate and severe disease, a history of documented mild COVID-19 was associated with lower levels of anti-RBD antibodies following vaccination, although no significant difference was observed in neutralizing antibody titers.

The findings of this study corroborate previous research demonstrating higher antibody titers among COVID-19 patients with severe or critical disease [22]. In this context, high antibody levels have been suggested to serve as a marker of COVID-19 severity since they reflect a state of increased viral replication, hyperinflammation, and activation of the cytokine storm [23]. On the other hand, despite higher measured antibody titers, severely ill patients have been shown to present significantly diminished anti-RBD neutralization potency [24]. Critical disease has been linked to the circulation of antibodies with lower binding affinity to the RBD and the nucleocapsid protein, suggesting an exaggerated but ineffective humoral response against SARS-CoV-2 [25]. Importantly, isotype analyses have indicated that patients developing severe COVID-19 present a significantly higher IgM and IgA response, while individuals with mild disease show an immunodominant IgG profile with greater immunological diversity, antibody class switching, and higher affinity, especially to the prefusion spike protein [26].

Previous studies have proposed the persistence of protective immunity in the mid-term following SARS-CoV-2 infection, which is in line with our observations. Abu-Raddad et al. [27] demonstrated the protective effects of antibody positivity for reinfection up to 7 months, while Egbert et al. [28] showed the durability of SARS-CoV-2 spike antibodies for up to 10 months after the initial infection. Terpos et al. [17] suggested that anti-RBD antibodies presented better longevity, although their ability for neutralization attenuated in a cohort of 143 individuals after a median of 8.3 months after their first COVID-19 symptoms. The levels of neutralizing antibodies have also been proposed to remain stable for at least 6 months after COVID-19 diagnosis, being higher for individuals with a history of more severe disease [29]. Interestingly, the durability of the immune response has been suggested to be elicited by the induction of both long-lived quiescent bone marrow plasma cells and memory B-cells, serving as persistent sources of antibodies against SARS-CoV-2 [30].

COVID-19 vaccination provides significant protection against severe disease, although its efficacy in preventing infection wanes by 30% after 6 months [31]. The titer of neutralizing antibodies has been shown to be significantly higher in vaccinated than in convalescent unvaccinated individuals [32]. The analysis of the Stop the Spread Ottawa cohort suggested that vaccination is safe and leads to an immediate augmentation of antibody titers among individuals with prior infection [33], while booster vaccines have been shown to provide protection against severe disease even in the Omicron variant infectious period [34]. The results of a retrospective study have indicated that the receipt of a single vaccine dose in patients with a history of natural COVID-19 effectively decreases the risk of both reinfection and symptomatic disease [35]. Similarly, Hammerman et al. [36] suggested that vaccination with the BNT162b2 vaccine among recovered individuals significantly reduces the risk of reinfection, especially in those younger than 65 years, irrespective of whether one or two doses were administered. Our findings are also in line with the SIREN study [37], which demonstrated that infection-acquired immunity remained high in individuals with subsequent vaccination, while it waned one year after natural infection among those unvaccinated. Additionally, the outcomes of a longitudinal study proposed that the receipt of two BNT162b2 doses in recovered individuals significantly boosted IgG spike and nucleocapsid antibodies, while a progressive decline in antibody levels was observed in cases of no vaccination [38].

The present study has several strengths. A prospective longitudinal design was implemented, allowing multiple antibody measurements throughout the year following the COVID-19 diagnosis. The characteristics of participants were captured in detail, providing an accurate categorization of COVID-19 severity based on clinical presentation, disease course, and patient outcomes. Both anti-RBD and neutralizing antibodies were evaluated, allowing a comprehensive profiling of immune responses before and after vaccination. Adjusted models were constructed, aiming to take into account the effects of potential confounders such as demographic characteristics and comorbidities.

On the other hand, the interpretation of outcomes may be limited by the moderate sample size and the inherent risk of observational studies for residual confounding. In addition, all patients were infected before the surge of the Omicron strain; thus, any possible differentiation of immune responses with newer variants of concern could not be evaluated. No breakthrough infections were observed during the follow-up; hence, the risk of reinfection could not be analyzed. A longer follow-up period might have detected events of reinfection, and it would be interesting to correlate the risk of reinfection with the antibody kinetics in time. It has to be noted that measures for the prevention of SARS-CoV-2 transmission were implemented in Greece during the study period. This may also explain the absence of reinfection events in our cohort. Furthermore, symptoms of long COVID were not evaluated in the context of the present study. The precision of estimates was limited by the available sample size, resulting in wide confidence intervals, especially regarding antibody levels following vaccination. It should also be acknowledged that few recovered individuals were fully vaccinated, precluding the drawing of safe conclusions regarding the effect of the number of vaccine doses on antibody levels.

In conclusion, this prospective cohort study suggests that severe COVID-19 elicits a strong immune response, as reflected by the persistently high levels of anti-RBD and neutralizing antibodies at 3 to 6 months after infection. Vaccination of recovered individuals leads to a significant augmentation in antibody titers, irrespective of disease severity. Further studies are needed to fully elucidate the long-term protective efficacy of SARS-CoV-2 antibodies against novel variants of concern, as well as to clarify the optimal vaccination strategy depending on the severity of prior COVID-19.

## Figures and Tables

**Table 1:** Baseline clinical and demographic characteristics of patients according to disease severity.

Variable	Overall (N = 106)	COVID-19 Severity			
		Mild (N = 28)	Moderate (N = 38)	Severe (N = 40)	*p*-Value
Age (years)	52 [42.3–59]	42.5 [32–53]	53 [45.3–59]	56 [50–59.5]	**0.001** ^a^
Male sex	71 (67.0)	15 (53.6)	25 (65.8)	31 (77.5)	0.116 ^b^
Caucasian ethnicity	97 (91.5)	26 (92.9)	35 (92.1)	36 (90.0)	1 ^b^
Hypertension	24 (22.6)	5 (17.9)	7 (18.4)	12 (30.0)	0.370 ^b^
Diabetes mellitus	15 (14.2)	1 (3.6)	2 (5.3)	12 (30.0)	**0.002** ^c^
Dyslipidemia	29 (27.4)	3 (10.7)	12 (31.6)	14 (35.0)	0.067 ^c^
Heart disease	8 (7.5)	1 (3.6)	1 (2.6)	6 (15.0)	0.106 ^c^
Respiratory disease	12 (11.3)	1 (3.6)	3 (7.9)	8 (20.0)	0.104 ^c^
Malignancy	1 (0.9)	0 (0.0)	0 (0.0)	1 (2.5)	1 ^c^
Charlson Comorbidity Index	1 [0–2]	0 [0–1]	1 [0–2]	2 [1–2.25]	**0.003** ^a^
Obesity	34 (32.1)	3 (10.7)	12 (31.6)	19 (47.5)	**0.006** ^c^
Smoking					
Former	35 (33.0)	3 (10.7)	16 (42.1)	16 (40.0)	**0.036** ^c^
Current	10 (9.4)	3 (10.7)	4 (10.5)	3 (7.5)	
Days from symptom onset	10 [8–13]	8.5 [7.8–12]	11 [8–13]	10 [8–13]	0.477 ^a^
Fever	101 (95.3)	23 (82.1)	38 (100)	40 (100)	**<0.001** ^b^
Cough	71 (67.0)	16 (57.1)	27 (71.1)	28 (70.0)	0.433 ^b^
Dyspnea	25 (23.6)	3 (10.7)	5 (13.2)	17 (42.5)	**0.002** ^b^
Chest pain	16 (15.1)	6 (21.4)	6 (15.8)	4 (10.0)	0.430 ^c^
Anosmia/Ageusia	23 (21.7)	5 (17.9)	5 (13.2)	13 (32.5)	0.099 ^b^
Diarrhea	24 (22.6)	6 (21.4)	7 (18.4)	11 (27.5)	0.622 ^b^
PSI/PORT score	68 [51–78]	52 [41.5–67.5]	66.5 [47.5–74]	78 [60.75–89.5]	**<0.001** ^a^
Remdesivir	84 (79.2)	11 (39.3)	34 (89.5)	39 (97.5)	**<0.001** ^b^
Corticosteroids	80 (75.5)	5 (17.9)	35 (92.1)	40 (100)	**<0.001** ^b^
Antibiotics	57 (53.8)	5 (17.9)	23 (60.5)	29 (72.5)	**<0.001** ^b^
Hospital stay (days)	11 [7–14]	6 [5,6]	10 [7.25–11]	14 [12–19.5]	**<0.001** ^a^
ICU admission	6 (5.7)	0 (0.0)	0 (0.0)	6 (15.0)	**0.006** ^c^
Mechanical ventilation	4 (3.8)	0 (0.0)	0 (0.0)	4 (10.0)	**0.037** ^c^
Death	2 (1.9)	0 (0.0)	0 (0.0)	2 (5.0)	0.334 ^c^
**Vaccination**	58 (54.7)	15 (53.6)	23 (60.5)	20 (50.0)	0.640 ^b^
1st dose	50 (47.2)	11 (39.3)	23 (60.5)	16 (40.0)	0.078 ^c^
2nd dose	8 (7.5)	4 (14.3)	0 (0.0)	4 (10.0)	
Type of vaccine					
BNT162b2	48 (45.3)	14 (50.0)	19 (50.0)	15 (37.5)	0.133 ^c^
mRNA-1273	7 (6.6)	0 (0.0)	4 (10.5)	3 (7.5)	
ChAdOx1	2 (1.9)	0 (0.0)	0 (0.0)	2 (5.0)	
Ad26.COV2-S	1 (0.9)	1 (3.6)	0 (0.0)	0 (0.0)	
Time from infection (months)	6.4 [5.7–6.8]	5.9 [5.0–6.5]	6.5 [5.9–7.6]	6.4 [5.7–6.8]	0.162 ^a^

Data presented as median [interquartile range] or number (column percentage). Bold text indicates statistical significance. ^a^ Kruskal–Wallis test; ^b^ chi-square test; ^c^ Fisher’s exact test.

**Table 2:** Outcomes of antibody levels among patients with mild, moderate, and severe COVID-19.

Outcome	Overall	COVID-19 Severity			
		Mild	Moderate	Severe	*p*-Value
**Neutralizing antibodies without vaccination**					
Admission	28.01 [13.95–58.64]	18.65 [1.69–45.32] N = 11	34.57 [13.49–64.49] N = 35	29.63 [20.02–60.32] N = 37	0.190
Day 7	84.85 [60.51–89.76]	53.18 [40.32–72.46] N = 16	85.54 [62.27–88.71] N = 35	88.47 [80.69–91.48] N = 37	**0.001**
Day 30	89.38 [77.49–94.65]	77.56 [56.45–85.92] N = 27	92.00 [82.33–95.01] N = 38	91.90 [85.81–95.44] N = 39	**<0.001**
Month 3	86.40 [74.03–94.23]	66.92 [54.56–83.56] N = 24	85.80 [79.09–92.86] N = 36	94.21 [86.46–96.28] N = 31	**<0.001**
Month 6	80.80 [69.93–90.89]	75.96 [64.18–89.02] N = 19	78.13 [62.82–89.89] N = 19	90.23 [76.65–94.27] N = 24	**0.026**
Month 12	73.35 [46.90–87.03]	14.6 [14.6–14.6] N = 1	90.05 [81.89–96.35] N = 4	49.72 [44.08–60.43] N = 3	0.054
**Anti-RBD antibodies without vaccination**					
Admission	0.73 [0.40–8.38]	0.40 [0.40–5.46] N = 18	2.00 [0.40–13.39] N = 35	0.76 [0.40–3.48] N = 37	0.160
Day 7	67.62 [8.49–162.23]	8.26 [1.05–25.92] N = 16	93.00 [7.55–165.03] N = 34	106.15 [29.89–173.70] N = 38	**0.004**
Day 30	277.25 [104.36–499.73]	99.13 [25.54–207.95] N = 27	352.55 [197.57–462.73] N = 38	463.90 [186.90–717.75] N = 39	**<0.001**
Month 3	419.40 [169.30–711.50]	181.80 [54.23–294.80] N = 23	426.95 [230.10–550.65] N = 36	747.05 [276.20–1381.00] N = 30	**<0.001**
Month 6	417.75 [184.68–662.60]	348.60 [177.05–631.35] N = 19	414.10 [219.40–532.70] N = 29	558.30 [256.07–1141.50] N = 24	0.151
Month 12	518.60 [130.36–1271.28]	4339.36 [2193.04–6485.86] N = 2	542.4 [518.6–1914.2] N = 3	158.1 [102.61–378.90] N = 3	0.707
**Neutralizing antibodies after vaccination**					
Month 6	97.59 [96.72–97.85]	96.59 [94.76–97.77] N = 6	97.81 [96.82–97.86] N = 5	97.59 [97.42–97.86] N = 7	0.584
Month 12	97.32 [96.88–97.62]	97.11 [96.65–97.52] N = 12	97.33 [96.89–97.47] N = 20	97.37 [96.98–97.85] N = 15	0.496
**Anti-RBD antibodies after vaccination**					
Month 6	14,858 [19–839,839–2008]	2150 [10–252,252–1263] N = 6	18,262 [22–550,550–5982] N = 5	17,367 [17,21–114,184] N = 3	0.140
Month 12	8800 [4392.5–12,912]	3379 [2532–8198.5] N = 12	11,107 [14–206,206–5846] N = 9	9816 [17–68,68–7392] N = 16	**0.004**

Data presented as median [interquartile range]. Comparisons were performed with the Kruskal–Wallis test. Bold text indicates statistical significance.

**Table 3:** Linear regression analysis of the association between disease severity and antibody levels in different timepoints.

Outcome	Unadjusted Model		Age- and Sex-Adjusted Model		Fully Adjusted Model	
	Moderate Disease	Severe Disease	Moderate Disease	Severe Disease	Moderate Disease	Severe Disease
**Neutralizing antibodies**						
Admission	11.32 (−5.31; 27.94)	9.60 (−6.87; 26.07)	8.18 (−9.63; 25.99)	5.27 (−13.10; 23.64)	6.69 (−12.05; 25.42) *R*^2^: 0.04	4.46 (−15.02; 23.95)
Day 7	**14.87 (1.68; 28.05)**	**25.54 (12.47; 38.61)**	13.03 (−0.81; 26.87)	**22.99 (8.66; 37.32)**	11.07 (−3.19; 25.32) *R*^2^: 0.21	**23.02 (8.17; 37.88)**
Day 30	**18.13 (10.15; 26.11)**	**19.58 (11.65; 27.52)**	**15.59 (7.33; 23.85)**	**16.72 (8.12; 25.31)**	**13.87 (6.05; 21.70)** *R*^2^: 0.40	**18.26 (9.87; 26.65)**
3rd month	**15.19 (6.89; 23.50)**	**22.87 (14.30; 31.44)**	**13.02 (4.47; 21.57)**	**20.31 (11.11; 29.51)**	**12.44 (3.96; 20.92)** *R*^2^: 0.35	**21.47 (12.04; 30.90)**
**Anti-RBD antibodies**						
Admission	64.87 (−59.91; 189.65)	22.69 (−100.94; 146.31)	27.22 (−103.41; 157.85)	−28.84 (−163.41; 105.73)	47.77 (−88.31; 183.85) *R*^2^: 0.07	−7.97 (−149.80; 133.86)
Day 7	72.42 (−44.70; 189.53)	112.10 (−3.02; 227.23)	54.08 (−68.17; 176.34)	85.67 (−68.17; 176.34)	32.11 (−97.70; 161.94) *R*^2^: 0.07	69.43 (−63.93; 202.79)
Day 30	271.19 (−9.32; 551.69)	**525.61 (246.61; 804.62)**	197.80 (−97.00; 492.60)	**438.95 (132.25; 745.65)**	200.66 (−109.06; 510.38) *R*^2^: 0.15	**427.35 (95.32; 759.37)**
3rd month	215.16 (−35.42; 465.73)	**631.62 (371.46; 891.78)**	178.28 (−84.42; 440.48)	**586.61 (301.93; 870.69)**	159.83 (−113.65; 433.30) *R*^2^: 0.25	**563.09 (257.02; 869.17)**

Data presented as *β* (95% confidence intervals). Mild disease serves as the reference group. Bold text indicates statistical significance. The fully adjusted model adjusts for age, sex, ethnicity, obesity, and Charlson Comorbidity Index.

**Table 4:** Linear regression analysis of the association between disease severity and antibody levels after vaccination.

Outcome	Unadjusted Model		Age- and Sex-Adjusted Model		Fully Adjusted Model	
	Moderate Disease	Severe Disease	Moderate Disease	Severe Disease	Moderate Disease	Severe Disease
**Neutralizing antibodies**						
Month 6	1.06 (−0.39; 2.41)	1.23 (−0.06; 2.51)	0.99 (−0.80; 2.77)	1.16 (−0.43; 2.76)	1.44 (−1.73; 4.62) *R*^2^: 0.50	1.50 (−1.29; 4.29)
Month 12	1.81 (−0.59; 4.22)	2.05 (−0.50; 4.59)	1.68 (−0.83; 4.18)	1.93 (−0.81; 4.67)	1.10 (−1.87; 4.07) *R*^2^: 0.17	1.04 (−2.23; 4.30)
**Anti-RBD antibodies**						
Month 6	8186.33 (−3276.43; 19,649.10)	13,280.67 (−104.96; 26,666.29)	6291.59 (−9978.57; 22,561.76)	11,820.99 (−4140.53; 27,782.52)	6007.56 (−9479.11; 21,494.23) *R*^2^: 0.85	9686.75 (−5563.35; 24,936.86)
Month 12	**6591.63** **(1786.05; 11,397.21)**	**8137.58** **(3160.65; 13,114.50)**	**6185.36** **(1229.85; 11,140.87)**	**7266.22** **(1956.65; 12,575.80)**	**5615.19** **(657.92; 10,572.46)** *R*^2^: 0.41	3900.99 (−1519.85; 9321.84)

Data presented as *β* (95% confidence intervals). Mild disease serves as the reference group. Bold text indicates statistical significance. The fully adjusted model adjusts for age, sex, vaccine type, number of vaccine doses, and interval from vaccination.

## Data Availability

Raw data are available upon reasonable request from the corresponding author.
